# Oral mucoceles: A Brazillian Multicenter Study of 1,901 Cases

**DOI:** 10.1590/0103-6440202204965

**Published:** 2022-10-21

**Authors:** Georgea Gabriela Barreto Miranda, Samuel de Carvalho Chaves-Junior, Matheus Paschoaletto Lopes, Talytha Barbosa da Rocha, Débora Frota Colares, Fábio Augusto Ito, Israel Leal Cavalcante, Roberta Barroso Cavalcante, Bruno Augusto Benevenuto de Andrade, Cassiano Francisco Weege Nonaka, Pollianna Muniz Alves, Ricardo Luiz Cavalcanti de Albuquerque-Júnior, John Lennon Silva Cunha

**Affiliations:** 1 Laboratory of Morphology and Experimental Pathology, Institute of Technology and Research, Tiradentes University(UNIT), Aracaju, Brazil; 2 Department of Pediatric Dentistry, Federal University of Para(UFPA), Belem, Brazil.; 3 Piracicaba Dental School, University of Campinas (UNICAMP), Piracicaba, Brazil.; 4 Postgraduate Program of Dentistry, Department of Dentistry, State University of Paraíba (UEPB), Campina Grande, Paraíba, Brazil; 5 Departament of Dentistry, Federal University of Rio Grande do Norte(UFRN), Natal, Brazil; 6 Department of Oral Medicine and Pediatric Dentistry, State University of Londrina (UEL), Londrina, Brazil; 7 School of Dentistry, University of Fortaleza(UNIFOR), Fortaleza, Brazil; 8 Department of Oral Diagnosis and Pathology, School of Dentistry, Federal University of Rio de Janeiro(UFRJ), Rio de Janeiro, Brazil

**Keywords:** Mucocele, Histopathology, Salivary gland, diagnosis

## Abstract

Oral mucocele (OM) is the most common lesion of minor salivary glands. The present study aimed to report the clinical and demographic features of a large series of OMs and identify possible predictive variables associated with the recurrence rate of these lesions. A retrospective descriptive cross-sectional study was performed. A total of 43,754 biopsy records from four pathology services in Brazil were analyzed. All cases of OMs were reviewed, and clinical and demographic data were collected. The study comprised 1,002 females (56.2%) and 782 males (43.8%), with a mean age of 19.8±16.4 years (range: 01-87 years) and a 1.3:1 female-to-male ratio. The lower lip (n=1,160; 67.4%), and floor of the mouth (n=172; 10.0%), were the most common affected sites, presenting clinically as nodules (n=978; 79.4%) of smooth surface (n=428; 77.5%) and normal color (n=768, 46.7%). Excisional biopsy was the treatment in most cases (n=1,392; 78.0%). Recurrent OMs represented 6.2% of all diagnosed cases (n=117). OMs recurred more commonly in younger patients (aged<20 years) (*p*<0.0001), in lesions larger than 2 cm in diameter (p<0.0001), and in those located in the ventral tongue (p=0.0351). Also, recurrence rates were higher significantly in cases treated with laser surgery than in those with conventional surgery (p=0.0005). Patients with OMs should be carefully informed of its possible recurrence, especially when found on the ventral tongue of young patients.

## Resumo

A mucocele oral (MO) é a lesão mais comum das glândulas salivares menores. O presente estudo teve como objetivo relatar as características clínicas e demográficas de uma grande série de MOs e identificar possíveis variáveis preditivas associadas à taxa de recorrência dessas lesões. Foi realizado um estudo transversal descritivo retrospectivo. Foram analisados 43.754 registros de biópsias de quatro serviços de patologia no Brasil. Todos os casos diagnosticados como MOs foram revisados e dados clínicos e demográficos foram coletados. Participaram do estudo 1.002 mulheres (56,2%) e 782 homens (43,8%), com média de idade de 19,8 ± 16,4 anos (variação: 01-87 anos) e proporção de mulheres para homens de 1,3:1. O lábio inferior (n=1.160; 67,4%) e assoalho da boca (n=172; 10,0%), foram os locais mais acometidos, apresentando-se clinicamente como nódulos (n=978; 79,4%) de superfície lisa (n =428; 77,5%) e coloração normal (n=768, 46,7%). A biópsia excisional foi o tratamento na maioria dos casos (n=1.392; 78,0%). As MOs recorrentes representaram 6,2% de todos os casos diagnosticados (n = 117). As recorrências recorreram mais comumente em pacientes mais jovens (idade < 20 anos) (p < 0,0001), em lesões maiores que 2 cm de diâmetro (p < 0,0001) e naquelas localizadas na superfície ventral da língua (p = 0,0351). Além disso, as taxas de recorrência foram significativamente maiores nos casos tratados com cirurgia a laser do que aqueles com cirurgia convencional utilizando bisturi (p = 0,0005). Pacientes com mucoceles devem ser informados sobre uma possível recorrência, principalmente quando encontrados no lábio ou assoalho bucal de pacientes jovens.

## Introduction

Mucoceles, also known as *mucus escape reaction* or *mucus extravasation phenomenon*, are common non-neoplastic lesions of the salivary glands ^1^ ^,^ ^2^. Oral mucoceles (OMs) are most commonly seen in children and young adults, with a peak incidence in the second decade of life ^1^ ^,^ ^2^. The lower lip is the most frequently affected site, followed by the floor of the mouth and ventral tongue ^1^ ^,^ ^2^ ^,^ ^3^. When it occurs on the floor of the mouth, the term *ranula* is used ^4^. Clinically, the lesions present with an asymptomatic dome-shaped nodule or blister, often with a blue hue due to the extravasated mucin ^1^ ^,^ ^2^ ^,^ ^3^.

The term "mucocele" should be reserved preferentially for lesions resulting from the rupture of a duct of the salivary glands and consequent extravasation of mucus to the surrounding connective tissue ^1^. Characteristic morphological findings include mucus extravasation associated with granulation tissue formation ^1^ ^,^ ^5^. According to this definition, mucoceles do not have a true cystic epithelial lining. On the other hand, it is preferable to use the term "salivary duct cyst" (SDC), also known as "sialocyst" or "mucus retention cyst," for cysts lined by salivary ductal epithelium resulting from an obstructive phenomenon of salivary glands ^1^ ^,^ ^6^. Nowadays, there is evidence that the mucus extravasation phenomenon and the salivary duct cyst are distinct clinicopathological entities ^1^ ^,^ ^6^. Therefore, in this study, we used the terms "mucocele" and "salivary duct cyst" according to these definitions.

Although several oral mucoceles studies have been published previously ^1^ ^,^ ^2^ ^,^ ^7^ ^,^ ^8^ ^,^ ^9^, there is still confusion in the terminology used to define these lesions. Some studies have used the term "mucocele" to refer to both the retention and extravasation phenomena ^8^ ^,^ ^10^ ^,^ ^11^ ^,^ ^12^ ^,^ ^13^. Other studies do not provide histopathological inclusion criteria ^14^. In addition, few reports have tried to determine the predictive variables for the recurrence of OM, and the results are controversial ^4^ ^,^ ^15^ ^,^ ^16^. Therefore, this study aims to report in detail the clinical and demographic findings of 1,901 cases of OMs and identify possible predictive variables associated with the recurrence rate of these lesions. To the best of our knowledge, this is the first Brazillian multi-institutional study with the largest sample of mucoceles in the oral cavity to date.

## Material and methods

### Ethical aspects

The Ethical Committee of the Tiradentes University (UNIT) (Protocol n º 3.238.266) approved the study.

### Study design and data obtaining

In this multi-institutional study, cases diagnosed as *mucus extravasation phenomenon* were retrieved from the archives of three Brazilian Oral Pathology Services and one Private General Pathology Service (Table 1). Data such as age, sex, ethnicity, anatomical location, size, symptoms, history of trauma and size variation, presence of multiple concurrent lesions, clinical diagnosis, treatment performed, and recurrence (when available) were obtained from clinical records and analyzed. Since the *ranulas* are considered a clinical variant of mucoceles ^2^, they also were included in this study. For cases in which the clinical diagnosis consisted of two or more diagnoses, we recorded the first diagnosis.

**Table 1:** Sources of the cases reviewed

Institution	State	Years	Lesions biopsied during the period studied	OM (%^a^)	%^b^
UNIFOR^c^	Fortaleza	1999-2019	16,977	863 (45.4)	5.1
UNIT^d^	Sergipe	2012-2019	1,083	66 (3.5)	6.1
PRIVATE PRACTICE^e^	Sergipe	2011-2019	3,876	178 (9.3)	4.6
UNIT^f^	Sergipe	1999-2007	21,818	794 (41.8)	3.6
Total	-	-	43,754	1,901 (100)	4.3

OM, oral mucocele; ^a^Percent concerning the number of cases of OM; ^b^Percent of the sample of OM at each center; ^c^Department of Dentistry, University of Fortaleza (North-East region); ^d^School of Dentistry, Tiradentes University (North-East region); ^e^Private practice of oral and maxillofacial pathology (CIMAGEM - Dental Imaging Center of Sergipe); ^f^Department of Medicine, *Nestor Piva Memorial,* Tiradentes University (North-East region).

**Figure 1:**
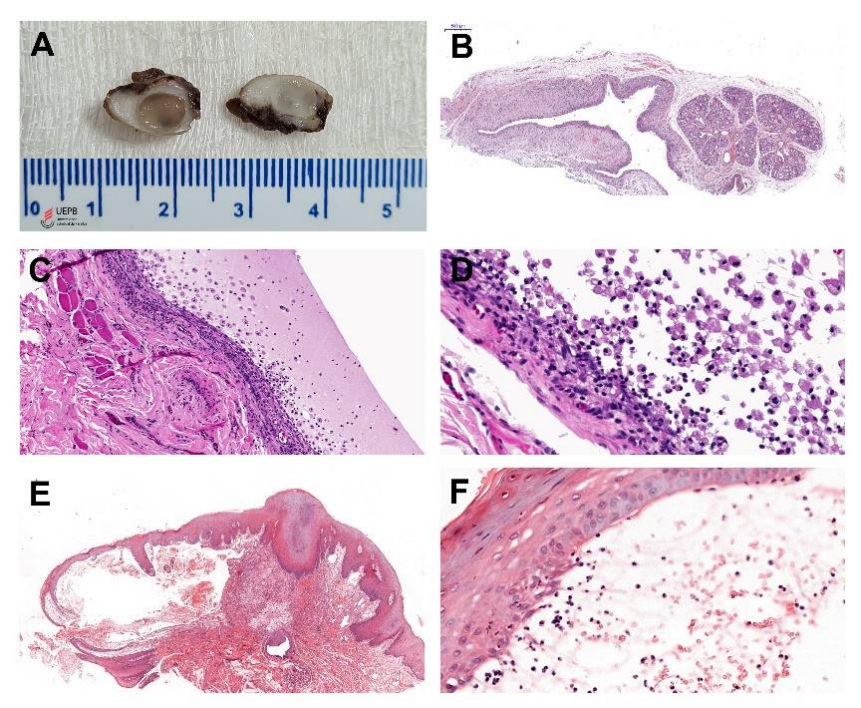
Gross and histopathological aspects of oral mucoceles. (A) The Gross section of the oral mucocele shows a lumen full of brown, gelatinous mucus. (B) Low power showing pathological cavity adjacent to the minor salivary glands. (C) Cavity covered by granulation tissue. (D). Detail of the numerous epithelioid foamy histiocytes (muciphages) inside the cavity and granulation tissue wall. (E and F) Superficial mucocele. Note a mucin-containing subepithelial blister (hematoxylin and eosin stain).

Clinically diagnostic lesions as superficial mucoceles were reevaluated morphologically. Five-micrometer hematoxylin and eosin-stained sections were obtained from each case, and two oral pathologists reevaluated the morphological features of the lesions. In cases of interobserver disagreement, the final diagnosis was reached by consensus. The diagnostic criteria of superficial mucocele included a mucin-containing subepithelial blister lined by atrophic surface epithelium (Figure 1).

### Analysis

Descriptive and quantitative analyses were performed using the Statistical Package for the Social Sciences for Windows 20.0 (SPSS, Inc., Chicago, IL, USA). Continuous variables were expressed as mean, median, and standard deviation values. Categorical variables were defined as the absolute number of cases and percentage values. Chi-square test and Fisher's exact test were used to evaluate the association between clinical and demographic characteristics, adopting a P‐value of ≤ 0.05 and 95% confidence interval.

## Results

In the present study, 1,901 cases were diagnosed as *mucus extravasation phenomenon*. The prevalence of *mucus extravasation phenomenon* was 4.3%, from 43,754 diagnostics. Among the 1,901 cases confirmed by histopathological analysis, 117 (6.2%) cases represented recurrences of previously biopsied lesions in the same anatomical site. The demographic and clinical data for the primary and recurrent oral mucoceles included in our analysis are summarized in Table 2.

**Figure 2:**
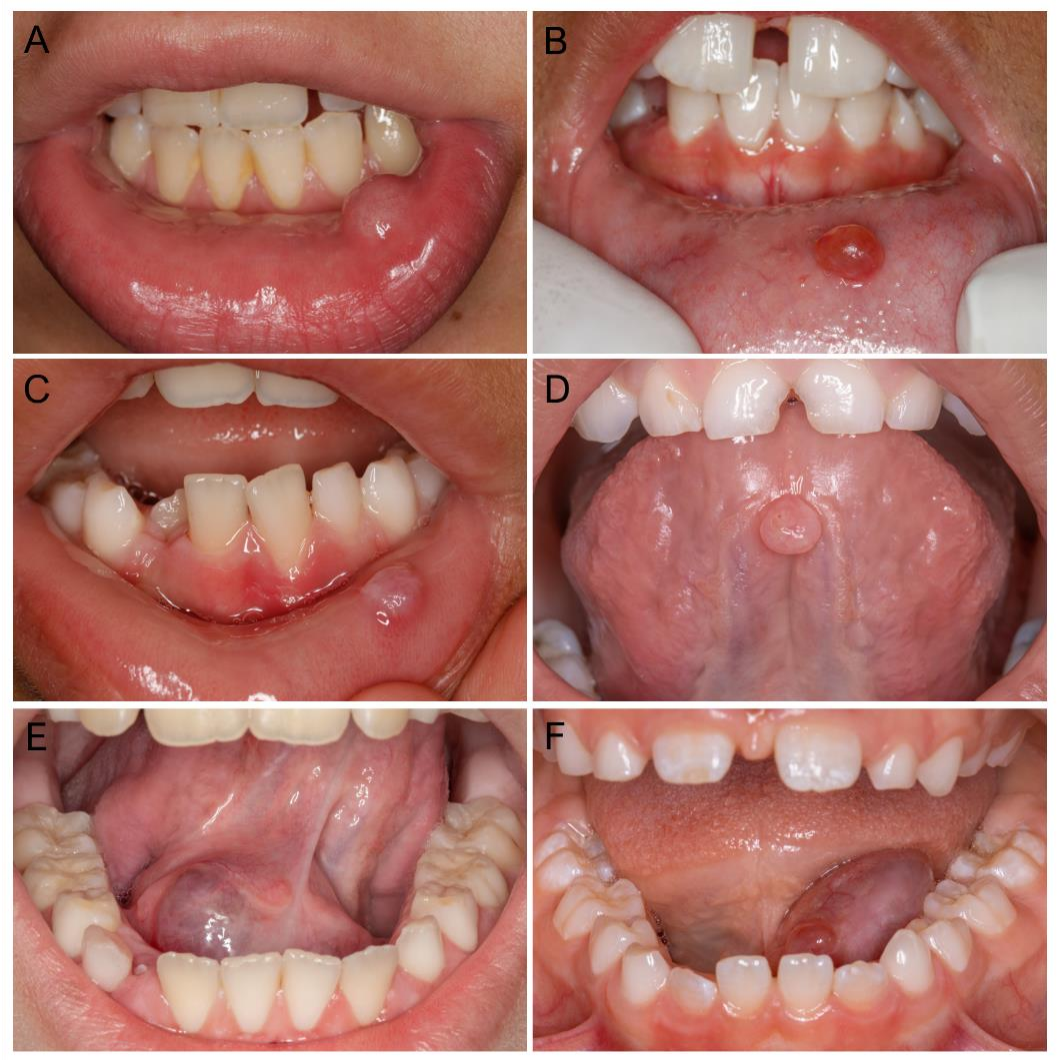
Clinical aspects of oral mucoceles (A-D) and ranulas (E and F). (A) Dome-shaped normochromic nodule in lower lip mucosa with the normal color surface. (B) Dome-shaped translucid reddish nodule in lower lip mucosa. (C) Submucous nodule in lower lip mucosa with a hyperkeratotic surface. probably due to secondary chronic trauma. (D) Pedunculated normochromic nodule in the ventral tongue. (E) Extensive submucous purplish swelling on the floor of the mouth. (F) Extensive exophytic blue-gray nodule with a smooth surface.

Oral mucoceles were more frequent in females (n = 1,002; 56.2%), with a mean age of 19.8 ± 16.4 years (ranging: 1‐87 years), and a 1.3:1 female‐to‐male ratio. Patients in the first (n = 426; 25.1%) and second (n = 602; 35.5%) decades of life were more affected, and most were Caucasian (n = 642; 52.5%). The lower lip was the most affected site (n = 1,160, 67.4%), followed by the floor of the mouth (n = 172; 10.0%), and the tongue (n = 166; 9.7%). Clinically, oral mucoceles presented as a submucosal well‐circumscribed nodule (n = 978; 79.4%) or blister (n = 168; 13.6%) with soft to elastic consistency. Most cases (n = 768; 46.7%) presented normochromic coloration and smooth surface (n = 428; 77.5%) (Figure 2). Only 38 cases (6.9%) showed ulceration at the time of the first consultation. Lesion size ranged from 0.5 to 6.0 cm, with a mean of 1.1 cm (SD ± 0.8). Most of them were asymptomatic (n = 1,250; 89.5%), although pain and discomfort had been mentioned in some cases (n = 146; 10.5%) (Table 2). The duration of the lesions varied from three days to two years (mean: 23.0 ± 31.1 weeks).

Interestingly, 38 cases (2.1%) were diagnosed as superficial mucoceles. However, only 9 of these cases (0.5%) met the clinical and microscopic criteria of superficial mucoceles. Of these, six cases (66.7%) occurred in women and three (33.3%) in men, with a mean age of 31.7 ± 10.1 years (ranging: 17‐48 years) and a 2:1 female‐to‐male ratio. The superficial mucoceles arose in the buccal mucosa (n = 5; 55.6%), lower lip mucosa (n = 2, 22.2%), and soft palate (n = 2; 22.2%). On the other hand, only one case (0.06%) of multiple mucoceles affecting the lower lip of a 7-year-old child was observed.

**Table 2:** Clinical and demographic findings of the present series of oral mucoceles.

Variables	Primary OM		Recurrent OM	
	(n)	(%)	(n)	(%)
*Age group (years)*				
0-9	426	25.1	41	36.0
10-19	602	35.5	57	50.0
20-29	378	22.3	11	9.6
30-39	156	9.2	5	4.4
40-49	34	2.0	0	0.0
50-59	40	2.4	0	0.0
60-69	42	2.5	0	0.0
70-79	14	0.8	0	0.0
80-89	6	0.4	0	0.0
NS	86	-	3	-
Range	1-87	-	5-36	-
Mean	19.8 ± 16.4	-	18.7 ± 13.6	-
*Sex*				
Female	1002	56.2	56	47.9
Male	782	43.8	61	52.1
F:M	1.3:1	-	1:1.1	
*Ethnicity*				
Caucasian	642	52.5	47	54.0
Afrodescendent	386	31.5	29	33.3
Brown	196	16.0	11	12.6
NS	560	-	30	-
*Anatomic location*				
Lower lip	1160	67.4	72	61.5
Floor of the mouth (ranula)	172	10.0	6	5.1
Tongue (ventral surface)	166	9.7	18	15.4
Others	86	5.0	8	6.8
Buccal mucosa	82	4.8	11	9.4
Palate	42	2.4	2	1.7
Upper lip	12	0.7	0	0.0
NS	64	-	0	-
*Type of lesion*				
Nodule	978	79.4	28	37.8
Blister (> 0.5 cm)	168	13.6	33	44.6
Polypoid mass	52	4.2	2	2.7
Vesicle (< 0.5 cm)	34	2.8	11	14.9
NS	552	-	43	-
*Surface*				
Smooth	428	77.5	62	63.3
Rough	86	15.6	27	27.6
Ulcerated	38	6.9	9	9.2
NS	1232	-	19	-
*Color*				
Similar to the mucosa	768	46.7	32	39.5
Translucent	288	17.5	28	34.6
Reddish	190	11.6	4	4.9
Bluish/Purplish	158	9.6	6	7.4
Whitish	156	9.5	11	13.6
Yellowish	26	1.6	0	0.0
Others	58	3.5	0	0.0
NS	140	-	36	-
*Size*				
Up to 2.0 cm	1122	85.6	49	57.0
> 2.0 cm	188	14.4	37	43.0
NS	474	-	31	-
Mean	1.1 ± 0.8	-	1.0 ± 0.9	-
Range	0.5-6.0	-	0.5-4.0	-
*Symptoms*				
Symptomatic	146	10.5	11	11.7
Asymptomatic	1250	89.5	83	88.3
NS	388	-	23	-
*Duration*				
< 6 months	113		61	52.1
≥ 6 months	92		56	47.9
NS	1579		-	-
Mean				
Range				
*History of trauma*				
Yes	422	77.3	12	70.6
No	124	22.7	5	29.4
NS	1218	-	102	-
*History of volume change*				
Yes	74	4.1	6	5.1
NS	1710	95.9	111	94.9
*Treatment*				
Scapel	1239	98.2	101	91.8
Laser	23	1.8	9	8.2

NS, not specified; OM, oral mucocele.

Regarding the clinical history, some patients reported previous trauma to the formation of the lesion (n = 442; 78.1%) and only 4.1% (n = 74) reported at the time of the consultation that they noticed the lesion showing variation in size during the clinical course. Regarding clinical diagnosis, 84.8% of cases (n = 1,450) were clinically diagnosed as mucoceles or ranulas. Other presumptive diagnoses included mainly reaction lesions, such as fibrous hyperplasia (n = 160; 9.4%), pyogenic granuloma (n = 28; 1.6%), squamous papilloma (n = 16; 0.9%) and cysts and benign tumors, including dermoid/epidermoid cysts (n = 24; 1.4%), lipoma (n = 14; 0.8%), hemangioma (n = 12; 0.7%), and pleomorphic adenoma (n = 6; 0.4%). Information on the hypothesis of clinical diagnosis was not available for 74 cases.

Most lesions were excised through excisional biopsy (n = 1,392; 78.0%). Surgical procedures were performed using a scalpel (n = 1,360; 97.7%) or CO_2_ laser (n = 23; 2.3%). There are 117 recurrent cases (6.2%) confirmed by histopathologic analysis. Analysis of recurrence rate according to clinical variables revealed significant differences depending on the anatomic site, age group, size, location, and treatment performed (Table 3). OMs recurred more commonly in younger patients (aged < 20 years) (p < 0.0001), in lesions larger than 2 cm in diameter (p < 0.0001) and in those located in the ventral surface of the tongue (p = 0.0351). Also, recurrence rates were significantly higher in cases treated with laser surgery than those with conventional surgery (p = 0.0005).

**Table 3:** Comparison of recurrence rate according to clinical data.

Variables	Number (Percentage)		***P* value**
	Primary OM	Recurrent OM	
*Sex*			0.4421*
Male	782 (93.3)	56 (6.7)	
Female	1002 (94.3)	61 (5.7)	
Age*			<0.0001*
Aged < mean	1028 (91.3)	98 (8.7)	
Aged ≥ mean	670 (97.2)	19 (2.8)	
*Location*			0.0351**
Lower and upper lip	1172 (94.2)	72 (5.8)	
Floor of the mouth (ranula)	172 (96.6)	6 (3.4)	
Tongue (ventral surface)	166 (90.2)	18 (9.8)	
Buccal mucosa/Palate	124 (91.9)	11 (8.1)	
Others	86 (89.6)	10 (10.4)	
*Ethinicity*			0.7256**
Caucasian	642 (91.8)	57 (8.2)	
Afrodescendent	386 (90.8)	39 (9.2)	
Brown	196 (90.3)	21 (9.7)	
*Size*			<0.0001*
Up to 2.0 cm	1122 (93.4)	79 (6.6)	
> 2.0 cm	188 (83.2)	38 (16.8)	
*Color*			0.0076**
Similar to the mucosa	768 (94.0)	49 (6.0)	
Translucent	288 (91.1)	28 (8.9)	
Reddish	190 (93.6)	13 (6.4)	
Bluish/Purplish	158 (94.6)	9 (5.4)	
Whitish/Yellowish	182 (96.3)	7 (3.7)	
Others	58 (84.1)	11 (15.9)	
*Surface*			0.6099**
Smooth	428 (83.1)	87 (16.9)	
Rough	86 (81.9)	19 (18.1)	
Ulcerated	38 (77.6)	11 (22.4)	
*Duration*			0.6427*
		
< 6 months	113 (64.9)	61 (35.1)	
≥ 6 months	92 (62.2)	56 (37.8)	
*Symptoms*			0.8745*
Symptomatic	146 (93.0)	11 (7.0)	
Asymptomatic	1250 (92.2)	106 (7.8)	
Treatment			0.0005*
Scapel	1239 (92.6)	101 (7.4)	
Laser	23 (71.9)	9 (28.1)	

Missing values were excluded; *Fisher's exact test; **Pearson Chi-square test. OM, oral mucocele.

## Discussion

The mucocele is the most common non-neoplastic lesion of salivary glands (1-3). Herein, we report the first Brazilian multi-institutional study with the largest case series of OMs reported to date. The mucoceles represented about 3.6% to 6.1% of the total lesions diagnosed in the referred pathology services in the present investigation, like previous studies ^1^ ^,^ ^2^. Although previous reports have reported male predominance ^1^ ^,^ ^3^ ^,^ ^5^ ^,^ ^9^, the present study revealed a slight female predominance (56.2%), with a female-to-male ratio of 1.3:1, consistent with other studies ^1^ ^,^ ^5^ ^,^ ^7^ ^,^ ^8^ ^,^ ^14^.

Oral mucoceles may occur at any age. However, it is most frequently seen in children and young adults (i.e., patients < 30 years old), with a peak incidence between 10 and 29 years ^1^ ^,^ ^2^ ^,^ ^5^ ^,^ ^7^ ^,^ ^8^, as seen in the present study. It is believed that people at this age are more prone to mechanical trauma, considered the main etiological factor for the mucus extravasation phenomenon ^1^ ^,^ ^2^ ^,^ ^3^. Trauma promotes rupture of the excretory duct of a salivary gland and the consequent extravasation of saliva to the adjacent connective tissue with the induction of an inflammatory reaction ^1^ ^,^ ^2^ ^,^ ^5^. In the present study, although most cases lack information about trauma history, most of the patients with available information reported local trauma before the lesion formation (77.3%).

Regarding the anatomical location, the lower lip mucosa (67.4%) was the most affected site, followed by the floor of the mouth, ventral tongue, and buccal mucosa. Similarly, most previous studies have reported the lower lip as the most common site, with the buccal mucosa, the floor of the mouth, and tongue as other frequent sites ^1^ ^,^ ^2^ ^,^ ^3^ ^,^ ^9^. However, any oral cavity location with minor salivary glands may be affected ^1^ ^,^ ^2^ ^,^ ^7^. The high incidence of mucoceles in the lower labial mucosa is not apparent. However, the propensity of the lips to mechanical chronic and acute trauma is the most accepted hypothesis for this incidence. Several studies have proposed that parafunctional habits (such as biting the lips), differences in the mobility of the upper and lower lips, or differences in the salivary glands' density are the leading causes ^1^ ^,^ ^2^. Also, the habit of sucking the lips and the use of piercing is eventually associated with oral mucoceles development.

Clinically, oral mucoceles appear as small floating dome-shaped nodules or blisters that may be slightly bluish, translucent, or normochromic ^1^ ^,^ ^2^ ^,^ ^3^ ^,^ ^5^ ^,^ ^7^ ^,^ ^8^, as observed in the present study (Table 2). The lesions' size varies between 5 and 9 mm and rarely reaches more than 1.5 cm in diameter ^1^ ^,^ ^2^ ^,^ ^3^ ^,^ ^7^. The duration may vary from a few days to several years ^2^. The lesions often spontaneously rupture and recur repeatedly due to the continued accumulation of mucus and secondary trauma ^2^ ^,^ ^16^. However, in the present study, only 4.1% (n = 74) of the patients reported that the lesion varied in size during the clinical course.

Similarly, the oral ranula presents clinically as a painless swelling on the floor of the mouth, bluish or translucent, and soft or mobile consistency on palpation ^17^. Deeper lesions may be normal in color. Generally, the ranula is dome-shaped, located laterally to the midline, with 2-3 cm in diameter ^17^, similar to the present study. However, sometimes it extends throughout the floor of the mouth and makes it difficult to position the tongue ^17^ ^,^ ^18^.

Some less frequent presentations of mucoceles such as congenital, superficial, and multiple can also be seen. Congenital mucoceles are rare lesions that develop during the intrauterine phase and mainly affect the lower lip ^19^. Superficial mucoceles are a distinctive form of lesion and can resemble mucocutaneous diseases ^1^ ^,^ ^2^ ^,^ ^20^. Interestingly, only nine patients (0.5%) of the present study met the clinical and microscopic criteria defined for superficial mucoceles ^20^. However, other authors believe the correct frequency was likely greater since superficial mucoceles are often not biopsied due to their transitory nature ^1^. These lesions tend to rupture spontaneously, leaving small superficial ulcers ^1^ ^,^ ^20^. In our study, most superficial mucocele cases occurred in women with a mean age of 31.7 years. They arose in the buccal mucosa, soft palate, and lower lip, similar to previous reports ^1^ ^,^ ^2^ ^,^ ^20^. Although the prevalence of superficial mucoceles found in this study is similar to previous reports ^1^, unfortunately, it does not accurately reflect the real prevalence of the sample since only cases clinically diagnosticated as superficial mucoceles were reassessed morphologically. The etiology of superficial mucoceles is controversial and remains unclear. However, superficial mucoceles have been associated with graft-versus-host-disease, mucous membrane pemphigoid, and lichen planus ^1^ ^,^ ^2^ ^,^ ^20^. They were also recently described as an oral complication post head and neck radiotherapy ^21^. Nevertheless, such associations were not observed in the present investigation.

Additionally, multiple mucoceles are also a rare form of lesions when seen in healthy patients ^22^. In our series, only one case of various mucoceles affecting the lower lip of a 7-year-old child was observed. The patient had a habit of biting and sucking the lower lip.

Oral mucoceles are diagnosed appropriately through clinical examination ^1^ ^,^ ^2^. In the present study, 84.8% of the cases were clinically diagnosed as mucoceles or ranulas. However, several benign and malignant conditions that affect the oral cavity can mimic mucoceles' clinical appearance ^2^ ^,^ ^22^ ^,^ ^23^. Thus, it is essential to submit the surgical specimen for histopathological analysis to confirm the diagnosis and rule out other pathologies that may have a worse prognosis ^22^. In addition, deeper lesions on the floor of the mouth usually have a similar color to the mucosa. They may recall pathologies such as dermoid/epidermoid cyst, thyroglossal duct cyst, branchial cyst hygromas, lipomas, and hemangiomas ^2^ ^,^ ^16^ ^,^ ^24^. In these circumstances, imaging tests are often necessary to assess the increase in cervical swellings ^2^ ^,^ ^24^. Ultrasonography is the most frequently used method in suspected plunging ranula cases. However, computed tomography or magnetic resonance imaging can also help assess glandular involvement and delimit the extent of the lesion ^24^. The lesion's aspiration can also be helpful in suspected plunging ranulas, being rich in saliva ^24^.

Some previously published large case series include SDCs in their analysis of OMs ^9^. However, we excluded SDCs from our analysis due to their distinct clinical and pathological features ^1^ ^,^ ^16^. SDCs of the oral cavity are less common lesions and tend to develop in older patients than mucoceles. Also, unlike mucoceles, SDCs do not exhibit a preference marked by the lower lip ^1^ ^,^ ^16^. Although SDCs are much less common, it is unlikely that their inclusion in a combined analysis of OMs significantly alters the results. However, we separated the two types of lesions because they represent different clinical and pathological entities.

The ideal treatment is to excise the lesions with the surrounding minor salivary glands to prevent recurrences. As alternative treatment options, marsupialization, micromarsupialization, cryotherapy, ablation with the CO_2_ laser, and local steroid therapy have been reported ^1^ ^,^ ^2^ ^,^ ^7^ ^,^ ^16^. However, surgical excision is still the most used because of the most favorable results. In the current study, most cases were treated by surgical excision (excisional biopsy), followed by careful remotion of the surrounding minor salivary glands.

Although it was not possible to accurately determine the recurrence rates of mucoceles in the present study due to many cases with incomplete clinical descriptions and a lack of regular follow-up, 117 patients (6.2%) represented previous recurrences of biopsied lesions in the same anatomical site. The literature reports recurrence rates ranging from 2.8% to 18.0% of cases ^3^ ^,^ ^7^ ^,^ ^10^ ^,^ ^13^. The variation is possibly due to the different surgical approaches and treatments performed. The present study found a statistically significant difference in the recurrence rate between OMs treated by surgical procedures using scalpels and lasers (*p*=0.0005). Previous reports have shown that conventional excision using scalpels resulted in a higher recurrence rate than laser excision ^15^.

In contrast, other studies reported that the recurrence rate was not significantly different between these surgical procedures ^3^ ^,^ ^25^. Therefore, despite the growing popularity of lasers, their advantage in preventing the recurrence of OMs remains carefully determined. In addition, our study also showed that OMs recurred more commonly in the younger patients (aged < 20 years) (*p* < 0.0001). The reason is possibly due to the considerable prevalence of several common oral habits, especially in childhood and adolescence, such as lip/cheek biting and tongue thrusting ^3^. Finally, the size lesion also influenced the recurrent rates. The recurrence rate was higher and statistically significant in oral mucoceles with a size more than 2 cm in diameter (p < 0.0001) and located on the ventral surface of the tongue (p = 0.0351). We believe this may be mainly due to the deeper location of the Blandin-Nuhn glands, which can make complete excision of the affected glands difficult, possibly due to a poor view of the operative field. Furthermore, it has also been reported that mucoceles on the ventral surface of the tongue are usually lined with thinner walls, which increases the possibility of easier rupture during removal ^3^. A sudden rupture would also cause loss of anatomical references and consequent and a precise boundary of the lesion, making it difficult to verify the complete removal of the mucocele ^3^.

There are some inherent limitations to the current study, mainly due to its retrospective nature. First, some clinical data was missing in the clinical records in some cases (e.g., sex, age, location of the lesion, etc.). Also, the recurrence rates were not determined based on regular follow-up.

In conclusion, OMs are lesions that are commonly diagnosed at Oral and Maxillofacial Pathology services. Our results confirm previous studies' findings regarding oral mucoceles' main clinical and demographic features, including a marked preference for the lower labial mucosa of children and young adults. Lesion size, site, age group, and treatment performed seems to be associated with the possibility of recurrence. Therefore, young patients must be informed of the probability of relapses. In general, the diagnosis can be made during a routine intraoral examination. However, histopathological analysis of the surgical specimen should be performed to confirm the diagnosis and rule out more serious pathologies. 
